# Clinical Applications of Cardiac Computed Tomography: A Focused Review for the Clinical Cardiologists

**DOI:** 10.3390/jcdd12100375

**Published:** 2025-09-23

**Authors:** Christian Giovanni Camacho-Mondragon, Juan Carlos Ibarrola-Peña, Daniel Lira-Lozano, Carlos Jerjes-Sanchez, Erasmo De la Pena-Almaguer, Jose Gildardo Paredes-Vazquez

**Affiliations:** 1 Escuela de Medicina y Ciencias de la Salud, Tecnologico de Monterrey, Monterrey 64710, Nuevo Leon, Mexico; christiangcm@icloud.com (C.G.C.-M.); jcibarrola.md@gmail.com (J.C.I.-P.); dlira92@gmail.com (D.L.-L.); carlosjerjes@tec.mx (C.J.-S.); erasmopa@mac.com (E.D.l.P.-A.); 2 Instituto de Cardiología y Medicina Vascular, TecSalud, Escuela de Medicina y Ciencias de la Salud, Tecnológico de Monterrey, San Pedro Garza Garcia 64700, Nuevo Leon, Mexico

**Keywords:** coronary artery disease, cardiac computed tomography, structural heart disease

## Abstract

Cardiac computed tomography (CT) has become a cornerstone in the non-invasive evaluation and management of cardiovascular disease, offering clinicians detailed anatomical and functional information that directly influences patient care. This review focuses on three primary clinical applications: coronary artery calcium (CAC) scoring, coronary CT angiography (CCTA), and preprocedural planning for structural heart interventions. CAC quantification remains one of the most powerful prognostic tools for cardiovascular risk stratification, with robust evidence supporting its use in asymptomatic and selected symptomatic individuals. CCTA provides a high-resolution assessment of coronary anatomy and plaque characteristics, guiding both preventive and acute care strategies. In structural heart disease, CT is indispensable for accurate device sizing, procedural planning, and complication avoidance in interventions such as transcatheter valve replacement or repair. Beyond these core applications, cardiac CT supports the evaluation of pericardial, myocardial, aortic, and congenital heart disease, and plays a role in pulmonary embolism risk assessment. Technological innovations—including artificial intelligence, dual-energy imaging, and photon-counting CT—are enhancing image quality, reducing radiation exposure, and broadening the modality’s prognostic capabilities. Collectively, these advances are solidifying cardiac CT as an integrated diagnostic and planning tool with a significant impact on clinical decision-making and patient outcomes.

## 1. Introduction

Since its inception, computer tomography imaging has represented a breakthrough in medicine. The early application of this technology in cardiovascular disease, showcasing its usefulness, dates back to the early 1990s, with coronary calcium quantitation by Arthur S. Agatston representing a significant advancement [1]. Since then, the development of new technologies, such as electron beam CT and subsequently multidetector CT imaging, along with the introduction of ECG gating, has allowed for the non-invasive collection of three-dimensional, high-resolution anatomical and functional data of the heart, coronary arteries, and surrounding structures, with improved temporal and spatial resolution [2].

Initially used for detecting coronary artery calcium, coronary CT imaging has evolved to provide additional prognostic value about plaque burden, composition, and functional significance [3].

The scope of this review is to analyze the three major cardiac CT applications in clinical practice. These applications are as follows: (1) coronary artery calcium quantification (CAC); (2) coronary computed tomography angiography (CCTA); (3) evaluation and planning of structural heart disease interventions.

## 2. Coronary Artery Calcium Quantification

As was previously stated, coronary artery calcium (CAC) quantification was the first application of CT in cardiovascular disease. Despite certain limitations, due to its relatively wide availability, low cost, and its non-contrast nature, as well as its thoroughly documented prognostic value, it remains one of the most widely used tests in clinical practice to complete cardiovascular evaluation in primary prevention check-ups to this day [4].

### 2.1. Technique and Clinical Data

The assessment of coronary artery calcium (CAC) involves a non-contrast computed tomography (CT) scan, synchronized with electrocardiography (ECG) to quantify calcified coronary plaque burden. The procedure is rapid, typically completed in a single breath-hold, and does not require intravenous access, premedication, or special patient preparation [4].

Using contemporary imaging techniques, the radiation exposure is approximately 1 to 1.5 mSv, equivalent to the radiation of 2–3 mammograms or approximately 6 months of ambient background radiation [5]. After image acquisition, the total coronary calcium burden is quantified using the Agatston scoring method in a commercially available software, which is derived from the extent and density of calcified plaque within the coronary arteries [4].

Coronary calcium score is a continuous numerical variable. Studies suggest that it has a linear relationship to adverse clinical outcomes. The following classification is widely used as a mean of standardization. Classification: 0 (absent), 1–10 (minimal) 10–99 (mild), 100–399 (moderate), 400–1000 (severe), or >1000 (extreme), although gender and age-based sub-classifications have been shown to provide a more accurate prognostic value, particularly in individuals with scores classifying as “moderate” or young (<45) or older (>70) individuals, in which a score above the 75th percentile should be considered as significant in guiding primary prevention practices [1].

### 2.2. Rationale and Evidence of CAC Scoring in Cardiovascular Risk Assessment

Multiple algorithms have been proposed to help clinicians identify who is, and who is not, at high risk for CAD, such as SCORE-2 [6] (p2), SCORE-2 OP, Framingham risk scores (FRS), Pooled Cohort Equations, and carotid intima-media thickness, among others. However, coronary artery calcium (CAC) scoring consistently outperforms these tools. In the Multi-Ethnic Study of Atherosclerosis (MESA), involving 6814 asymptomatic adults across four ethnic groups followed for an average of 11 years, individuals with CAC > 300 had nearly a 10-fold increased risk of coronary heart disease (CHD) events compared with those with CAC = 0, even after adjusting for traditional risk factors [7].

The CAC Consortium, pooling over 66,000 asymptomatic individuals with a mean follow-up of ~12.3 years, demonstrated that annualized cardiovascular disease (CVD) mortality was 0.33 per 1000 person-years in patients with CAC = 0, compared to 8.0 per 1000 person-years in those with CAC ≥ 1000 (adjusted hazard ratio [HR] 5.0, 95% CI 3.9–6.5 versus CAC = 0; HR 1.7, 95% CI 1.4–2.1 versus CAC 400–999) [8].

The Dallas Heart Study, which included substantial representations of Black and Hispanic individuals, revealed that though Black participants had lower CAC scores on average, they still experienced disproportionately higher CVD event rates at any given CAC level, indicating potential disparities in calcium-risk thresholds [9].

In a post hoc analysis of the St. Francis Heart Study, a randomized trial of atorvastatin versus placebo in nearly 1000 asymptomatic individuals with elevated CAC (≥80th percentile by age/sex), a median follow-up of 4.8 years showed that untreated eligible participants with CAC > 300 experienced event rates ≥ 17 per 100 person-years, whereas those with CAC < 100 had rates < 1 per 100 person-years. Statin therapy significantly reduced events, and combining CAC with clinical risk improved net reclassification (*p* = 0.002) [10].

The Rotterdam Study, conducted in an elderly European cohort, further confirmed that CAC significantly enhances CHD risk prediction beyond traditional models in older adults [11].

While many studies have focused on using coronary artery calcium (CAC) scoring to identify high-risk patients, evidence suggests that the absence of coronary calcifications (CAC score of zero) is associated with an exceptionally low 10 year cardiovascular event rate. This finding is particularly significant in individuals with a borderline or intermediate atherosclerotic cardiovascular disease (ASCVD) 10 year risk, potentially reclassifying these individuals as low risk and thus aiding in clinical decision-making in regard to holding off or starting statin therapy, and despite current guidelines’ recommendation to test CAC scoring in patients 40–75 years old [12], younger patients represent another group in whom CAC scoring may provide relevant prognostic information; in a sub-analysis of the CAC consortium study, out of ~22,000 of adults aged 30 to 49 years undergoing CAC testing for clinical indications, 34% had detectable coronary calcium, and those with scores > 100 were found to have a 10× increased risk of CHD mortality compared to those of the same age with a CAC of 0 [7].

Furthermore, recent studies have suggested that patients with higher CACS increase the prediction of abnormal myocardial strain by CT [13], although robust longitudinal data are lacking.

### 2.3. Relevance of Coronary Calcium Distribution

In the CAC consortium study, the largest CAC cohort to date, ~66,000 participants with no coronary artery disease enrolled between 1991 and 2010; findings suggest that after adjusting for total CAC and baseline risk factors, those with left main lesions and proximal multi-vessel calcium distribution had 20–30% higher CVD mortality. A notable exception to this finding were patients with extreme CAC scores (>1000) who had a mortality rate per 1000 person-years of 8.0, compared to 3.6 for those with CAC scores of 400 to 999. It is, however, worth mentioning that these findings might be challenged by clinical data from other cohorts and prospective studies. For example, in the MESA (Multi-Ethnic Study of Atherosclerosis), patients with CAC scores >1000 were at no higher risk of CHD death or MI than those in the 400–999 group (though with a much smaller sample size and shorter follow-up) [7]. The CAC Data and Reporting System (CAC-DRS), a new, validated scoring system, uses both total Agatson score and number (0–4) of the major coronary arteries affected, and is it recommended by the SCCT/STR guidelines for coronary artery calcium scoring, and permits a standardized report to improve the communication with a clinical physician [14].

### 2.4. Relevance of Coronary Calcium Plaque Volume and Density

Another major caveat that clinicians should keep in mind when evaluating CAC scoring during an individual’s risk assessment is the fact that data from several studies using other imaging techniques suggest that denser calcified plaque may be more stable over time [15], and that statin therapy actually increases calcium deposition in atherosclerotic plaque, protecting against plaque destabilization [16].

This discordance in findings could be explained by the fact that the overall CAC score is directly dependent on both plaque area (volume-dependent) and plaque density (derived from Hounsfield units), and there might be different phenotypes within similar CAC scores, with some patients with severe or extreme CAC scores having increased plaque density but not plaque burden, which paradoxically might be associated with lower CVD risk. Criqui et al. tested this hypothesis using data derived from the MESA cohort and found that CAC density was inversely related to CVD events at a given CAC volume, and that volume was more predictive when adjusted for CAC density, although more research is needed to precisely clarify the relationship between plaque burden, distribution, and density with cardiovascular outcomes, and how this may differ between age and gender groups [7].

### 2.5. Guideline Recommendations of CAC Assessment

Most guidelines endorse CAC as a risk modifier in individuals at borderline to intermediate, asymptomatic ASCVD risk, but they differ in details such as the age range, risk calculation models, and thresholds for statin initiation. Importantly, a CAC score of zero was consistently associated with down-classification of risk and deferral of pharmacologic therapy, while elevated CAC (e.g., ≥100 Agatston units or above the 75th percentile) often led to treatment intensification [17]. 

The 2019 American College of Cardiology/American Heart Association (ACC/AHA) Guideline on the Primary Prevention of Cardiovascular Disease and the 2021 Canadian Cardiovascular Society Guidelines for the Management of Dyslipidemia recommend coronary artery calcium (CAC) scoring as a tool for refining risk assessment in adults aged ≥40 years with intermediate (7.5–20%) 10 year ASCVD risk, and also in selected patients at borderline risk. The ACC/AHA limits its benefit to individuals up to age 75. More recently, focused updates from the European guidelines highlight that while CAC is not recommended as a broad population screening test, its consideration improves both risk discrimination and reclassification. In particular, the presence of subclinical coronary atherosclerosis on imaging or an elevated CAC score should now be regarded as a risk modifier in individuals at moderate risk or in those around treatment decision thresholds, with a formal recommendation of Class IIa, Level of Evidence B [18].

**Figure 1:**
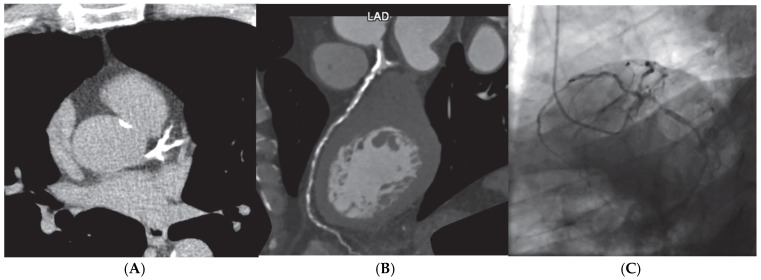
Screening finding of coronary calcium score of 2388 AU (**A**) on an asymptomatic patient leading to coronary computer cardiac tomography (CCTA) (**B**) showing extensive multivessel obstructive disease, which was then confirmed by (**C**) invasive coronary angiography.

In a recent study [19], 1903 statin-naïve adults aged 55–75 years without ASCVD or diabetes were chosen from the Rotterdam Study to assess the impact of guideline-directed coronary artery calcium (CAC) scoring on statin treatment recommendations. Using ACC/AHA (PCE and PREVENT) and ESC/EAS (SCORE2/SCORE2-OP) risk models, the authors determined eligibility for CAC scans, proportions of individuals reclassified after CAC, ASCVD incidence rates, and estimated 10 year numbers needed to treat (NNT10y). Results showed that ACC/AHA guidelines identified fewer CAC scan-eligible individuals (up to 18% of men and 12% of women by PCE) compared with ESC/EAS (≈45% of both sexes), but virtually all CAC-eligible participants under ACC/AHA were reclassified, while ESC/EAS reclassified about half. Event rates and NNT10y (11–26 with high-intensity statins) supported CAC’s ability to refine risk, with up-risked individuals showing incidence rates similar to high-risk groups, and those with CAC = 0 showing very low event rates, reinforcing CAC as a valuable risk-modifying tool in primary prevention. An example of the clinical impact of elevated CAC score on clinical decision making is presented on Figure 1. 

## 3. Coronary Computed Tomography Angiography (CCTA)

Coronary computed tomography angiography (CCTA) is a contrast-enhanced, ECG-gated imaging modality used to detect and quantify both calcified and non-calcified coronary plaques. It also provides an estimation of the severity of luminal stenosis, aiding in the assessment of coronary artery disease severity and the high-risk factors to vulnerable plaques [3].

### 3.1. Technical Considerations and Contraindications

Before performing CCTA, an appropriate IV line (typically 18 gauge or 20 gauge in smaller patients) is needed to allow for rapid contrast injection (5–7 mL/s) during the scan [14]. Also, patients are often administered nitroglycerin to induce coronary vasodilation and beta-blockers (either orally or intravenously) to achieve lower heart rates, thereby optimizing image quality. The need for these medications varies depending on the type of CT scanner used, making pre-screening for contraindications essential (i.e., nitroglycerin contraindication in severe aortic stenosis) [3].

The scan is performed in a single breath-hold, typically lasting 5 to 10 s, and image acquisition occurs approximately 15 to 25 s after contrast injection; more precise timing can be determined using variations in bolus tracking, which monitors contrast attenuation in the ascending or descending aorta in real time [20].

Aside from a history of severe anaphylactic reaction to iodinated contrast, there are no absolute contraindications to CCTA [3]. However, certain scenarios require careful consideration to determine whether the potential benefits outweigh the risks; these include severe kidney impairment (eGFR < 30 mL/min/m^2^); uncontrolled tachycardia; severe obesity (BMI > 40); inability to comply with scan requirements (patients unable to follow breath-hold instructions or remain still during image acquisition); clinical instability, such as acute myocardial infarction or decompensated heart failure; pregnancy; presence of known severe, dense, calcific plaque or coronary stents with a diameter <3 mm which are challenging to evaluate due to limitations in spatial resolution; and the impact of blooming artifacts from metallic struts that can lead to overestimation of stenosis or render the segment non-assessable [20]. All of these should be assessed in an individual risk-to-benefit ratio on a case-by-case basis [12].

### 3.2. Safety and Radiation Exposure

The radiation dose in cardiac CT depends on several scan acquisition parameters. The tube voltage, measured in kilovolts (kV), determines the energy of the emitted photons, while the tube current, measured in milliamperes (mA) or milliampere-seconds (mAs), determines the number of photons produced [5,20]. Higher kV increases tissue penetration, whereas higher mA raises the total photon count, reaching the detector elements. Although increasing either kV or mA raises the radiation dose required for image acquisition, setting these values too low can result in excessive image noise and reduced diagnostic quality. Scanner settings should therefore be tailored to the patient’s body habitus and the desired image quality. For example, in obese patients, where higher noise levels and photon attenuation are expected, a higher kV is generally preferred.

Recent advances in coronary computed tomography angiography (CCTA) have enabled substantial reductions in radiation exposure while preserving diagnostic image quality. Key strategies include prospective ECG-gating to limit exposure to specific cardiac phases, and high-pitch helical acquisition with dual-source CT, allowing whole-heart imaging in a single cardiac cycle with doses often <1 mSv. Heart rate control with beta-blockers facilitates longer diastolic imaging windows, and limiting scan range minimizes unnecessary exposure. Advances in iterative reconstruction algorithms improve signal-to-noise ratios, enabling lower tube current and voltage settings without degrading the image quality [5]. The combination of these methods has made sub-millisievert CCTA feasible. A prospective multicenter registry demonstrated that over the past decade, the estimated radiation dose from cardiac CT has decreased by approximately 78%. Specifically, the median dose–length product (DLP) for CCTA fell from 885 mGy·cm in 2007 to 195 mGy·cm in 2017 [21].

### 3.3. Diagnostic Precision

Coronary angiography (CA) is considered the gold standard for the assessment of epicardial coronary anatomy, though it is an invasive test, and while infrequent, it is not exempt from serious complications (bleeding, vascular injury, and even myocardial infarction) [7].

Comparison of the diagnostic performance of CCTA versus coronary angiography has been assessed in several trials. Recently, a meta-analysis including nine studies found that CCTA had a sensitivity of ~96% and a specificity of ~75% to detect a stenosis of >50%. However, it can be argued that in clinical practice, identification of functionally significant stenoses is a more desired benchmark, the PACIFIC (Prospective Comparison of Cardiac PET/CT, SPECT/CT Perfusion Imaging and CT Coronary Angiography with Invasive Coronary Angiography) assessed the diagnostic performance of several imaging techniques compared to an invasively assessed FFR < 0.8, and they found CCTA had only moderate sensitivity and specificity (0.83 and 0.81) to detect functionally relevant stenosis [22,23].

To overcome this, novel techniques that allow the calculation of FFR (Fractional Flow Reserve) derived from CT have been developed. In the PACIFIC study, FFR-CT showed a sensitivity and specificity of 90% and 86%, respectively [23]. FFR-CT details are presented in Section 3.6.

### 3.4. Prognostic Information

A relevant question regarding the use of CCTA is whether imaging findings translate to adverse clinical outcomes, particularly when a plaque is considered non-significant, and whether these findings lead to a change in the medical management of these patients. Several studies have assessed this inquiry; in the SCOT-HEART (Scottish Computed Tomography of the HEART) trial, a prospective, multicenter, randomized trial of 4146 patients aged 18–75 years who had been referred by a primary-care physician to a dedicated cardiology clinic for patients with suspected stable angina due to coronary heart disease (CHD), patients were randomized 1:1 to receive either standard care plus CCTA or standard care alone. The patient population was intentionally broad to reflect a real-world setting and included a significant proportion of women (45%) and a median age of 57 years. The frequency of diagnosis of angina due to coronary artery disease (CAD) was lower, and diagnostic certainty was higher when CCTA was added, and nearly one in four patients in the CCTA group had their prescribed treatment altered at six weeks, compared with only one in twenty (5%) of those receiving standard care alone. Over a median follow-up of 4.8 years, the addition of CCTA to standard therapy reduced the composite endpoint of death from ischemic heart disease or nonfatal myocardial infarction by 41% (2.3% vs. 3.9%; HR 0.59; *p* = 0.004) [24].

While current CCTA technology and increased spatial resolution enable clinicians to diagnose and assess coronary atherosclerotic disease in its earliest stages, the main advantage of CCTA over coronary calcium is the ability to identify and categorize plaque features associated with acute coronary syndromes. These so-called “high-risk plaque features,” including low-attenuation plaque (which would be missed on standard calcium coronary assessment), positive remodeling (PR), spotty calcifications, and the napkin-ring sign have been linked to future acute coronary syndromes (ACS) [2]. Data from the SCOT-HEART and ICONIC studies show that these features are independent predictors of myocardial infarction (MI) even when stenosis severity is mild to moderate. For this reason, these high-risk features were added to the standardized report called CAD-RADS 2.0 [25].

In addition to plaque characterization, the non-invasive calculation of fractional flow reserve from CCTA (FFR-CT) has emerged as a valuable tool to functionally assess the hemodynamic significance of coronary lesions. The PACIFIC trial (Prediction of Advanced Coronary Artery Disease and Functional IsChEmia with CT) aimed to determine the diagnostic performance of FFR-CT compared to invasive FFR in 208 patients with suspected stable CAD, who underwent 256-slice coronary CTA, 99 mTc-tetrofosmin SPECT, [15O] H_2_O PET, and routine three-vessel invasive FFR measurements. The trial found that FFR-CT had the highest per-vessel diagnostic accuracy with a sensitivity of 90%, a specificity of 86%, and an AUC of 0.92. This was similar to PET, but superior to coronary CTA and SPECT. However, in a more clinically relevant intention-to-diagnose analysis that included the one in four unevaluable cases, FFR-CT’s performance was significantly diminished. On a per-vessel basis, its AUC was 0.83, no longer superior to CTA alone, and on a per-patient basis, its AUC of 0.79 was significantly outperformed by PET’s AUC of 0.90 [23].

Quantitative CCTA analysis has allowed researchers to monitor plaque progression and treatment response over time, such as the stabilization of non-calcified plaques with statin therapy in the PARADIGM study [26]. A major concern in the assessment of plaque burden and characterization is interobserver variability. Furthermore, automated software tools like vascuCAP [27] and QAngioCT [28] are enhancing accuracy and reducing interobserver variability by providing 3D plaque characterization.

### 3.5. Emerging CCTA Technologies

Beyond anatomy, emerging tools such as fractional flow reserve derived from CCTA (FFR-CT), perivascular fat attenuation index (FAI), and wall shear stress (WSS) [29] enable the functional assessment of lesions and provide deeper insight into plaque physiology and vascular inflammation. For instance, FAI correlates with coronary inflammation and may predict future adverse cardiovascular events [30].

In patients with high-intensity inflammatory diseases like HIV, psoriasis, rheumatoid arthritis, and SLE, CCTA has uncovered an increased prevalence of non-calcified plaques and vulnerable plaque features, which are often underdiagnosed using traditional modalities [31]. Longitudinal studies have shown that anti-inflammatory therapies can favorably modify plaque burden, further emphasizing the utility of CCTA in immune-mediated disease.

### 3.6. Functional Assessment

Currently, there are two CCTA technologies that enable the assessment of flow and coronary physiology: FFR-CT (Fractional Flow Reserve derived from Coronary CT Angiography) and CCTA perfusion imaging.

**Figure 2:**
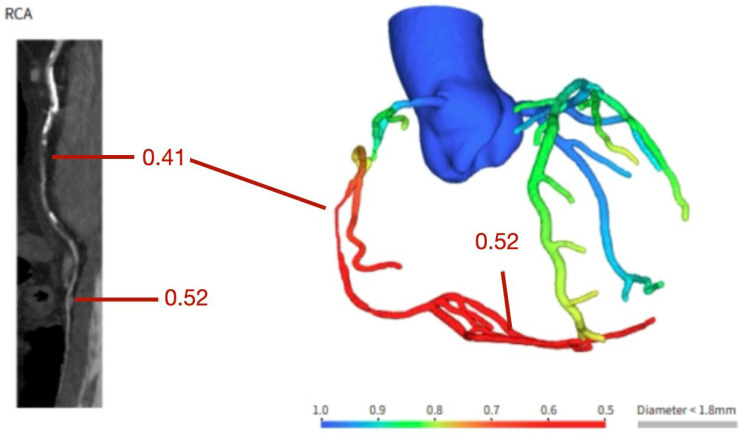
Coronary computer tomography fractional flow reserve assessment showing a functionally significant stenosis in the vertical segment of the right coronary artery.

FFR-CT is a non-invasive technique that allows for the estimation of three-vessel FFR values using standard CCTA datasets. Because FFR-CT is calculated directly from existing CCTA imaging data, it does not require additional testing or radiation exposure. FFR-CT calculations are based on computational fluid dynamics (CFD) models applied to CCTA data to estimate coronary blood flow parameters, including pressure gradients, flow velocity, and volumetric blood flow within the coronary arteries [32]. Threshold to significant stenosis is FFR-CT < 0.80 (Figure 2).

A key advantage of FFR-CT is its ability to provide a lesion-specific assessment of myocardial ischemia, thereby aiding in clinical decision-making regarding coronary revascularization. In the NXT trial (Analysis of Coronary Blood Flow Using CT Angiography: Next Steps) [22] a prospective, multicenter study that enrolled 254 patients referred for clinically indicated invasive coronary angiography, FFR-CT was performed on 484 coronary vessels and found that the area under the receiver operating characteristic (ROC) curve for FFR-CT was 0.90 per patient and 0.93 per vessel, corresponding to an overall per-vessel diagnostic accuracy of 86%, using invasive fractional flow reserve (FFR) as the reference standard [33].

Another functional tool is myocardial perfusion assessment in CCTA, which evaluates the distribution of iodinated contrast material during its first pass through the myocardium. Perfusion defects appear as hypoattenuating areas containing reduced amounts of contrast material. In static myocardial perfusion CT, assessment is often based on visual comparison of myocardial perfusion during pharmacological stress and at rest, which requires planning before the regular acquisition, since pharmacological stress images are not part of standard protocols. In contrast, dynamic myocardial perfusion CT acquires multiple sequential images during the first pass of contrast, enabling dedicated software to generate quantitative perfusion maps and objectively measure myocardial blood flow. With the advent of new multidetector CT scanners, both reproducibility and accuracy have increased, with reported sensitivity and specificity between 76 and 100% for static and dynamic CT perfusion imaging. This improvement offers incremental diagnostic value over coronary CCTA alone for the identification of hemodynamically significant CAD.

### 3.7. Impact of FFR-CT in Therapeutic Decision-Making

Several trials have assessed the impact of FFR-CT on clinical decision-making. The ADVANCE (Assessing Diagnostic Value of Non-invasive FFR-CT in Coronary Care) registry was a prospective, multicenter study that included 5083 patients across 38 centers, all of whom were referred for CCTA with FFR-CT assessment. The study found that incorporating FFR-CT into the CCTA workflow altered the initial treatment strategy in approximately two-thirds of cases. The authors also assessed the safety of deferring revascularization in patients with negative FFR-CT values (>0.80) [34]. Over a 90 day follow-up, none of the 1952 patients with negative FFR-CT experienced death, myocardial infarction (MI), unplanned hospitalization for acute coronary syndrome (ACS), or urgent revascularization. In contrast, 19 adverse events (ten deaths, four MIs, and five hospitalizations for urgent revascularization) occurred in the group with positive FFR-CT findings, yielding a crude risk ratio of 19.75 (*p* < 0.001) [34].

At one-year follow-up, there was a trend toward fewer major adverse cardiovascular events (MACE) in patients with negative FFR-CT (*p* = 0.062) and a significant reduction in cardiovascular death or MI compared to those with abnormal FFR-CT values (*p* = 0.01); specifically, patients with intermediate coronary stenosis who had a negative FFR-CT result (>0.80) demonstrated long-term clinical outcomes comparable to those with no or minimal stenosis (0–30%) on CCTA [34].

In the PROMISE trial, an observational cohort study that included 181 patients with stable chest pain, an FFR-CT value of ≤0.80 was found to be a significantly better predictor of revascularization or major adverse cardiovascular events (MACE) than severe (>70%) stenosis on computed tomography angiography (CTA). The authors concluded that FFR-CT could improve cost efficiency by enhancing the selection of patients referred for invasive coronary angiography [35].

However, it is relevant to emphasize that when FFR-CT is used to guide treatment strategies, the evaluation of other clinical and anatomical factors is paramount, such as the high-risk features discussed previously. This tool is particularly valuable for assessing moderate coronary artery stenoses, especially those found in the proximal or mid segments, where the significance of a lesion is not always clear from anatomy alone [32] because FFR-CT values tend to decline along the length of an artery; particularly in vessels with multiple narrowings or diffuse disease, it is essential to match areas of pressure drop with specific plaque locations because focal stenosis have the benefit of revascularization contrary to diffuse lesions [32]. This alignment is only possible by directly comparing CCTA images with the 3D FFR-CT model, which helps identify the lesion responsible for significant ischemia.

While several studies use a simple cutoff approach to interpret FFR-CT, it is important to remember that its values fall along a spectrum, with lower readings associated with higher risk for hemodynamic compromise and adverse events [34,35]. In cases where FFR-CT falls between 0.76 and 0.80, clinicians should integrate additional factors such as the exact lesion location, patient symptoms, presence of high-risk plaque features, and the magnitude of pressure drop across the lesion, to determine whether intervention is warranted [33].

FFR-CT presents several significant limitations. Currently, it requires dedicated technical personnel and incurs additional costs [32]. However, studies have demonstrated its cost-effectiveness by reducing the need for invasive coronary angiography and revascularization in selected patient populations [35]. Furthermore, the diagnostic reliability of FFR-CT is highly dependent on optimal image quality from the original CCTA. Artifacts such as motion, misalignment, low contrast resolution, or blooming from heavy coronary calcification can significantly impair accuracy [33]. Additionally, FFR-CT is not recommended for assessing stented coronary segments or in patients who have had prior coronary artery bypass graft (CABG) surgery, due to the limited validity of the method in those settings [35].

### 3.8. Results Reporting and Therapeutic Approach

**Figure 3:**
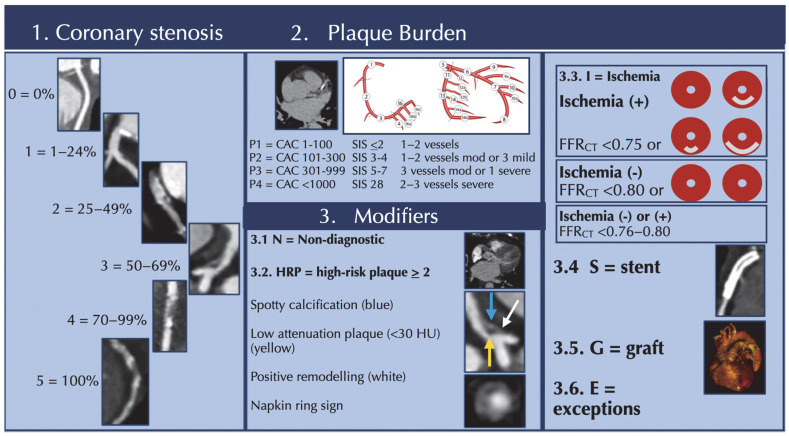
CAD-RADS 2.0 presented with examples and step-by-step configuration.

In 2022, an update to the expert consensus document by the Society of Cardiovascular Computed Tomography (SCCT), the American College of Cardiology (ACC), the American College of Radiology (ACR) and the North America Society of Cardiovascular Imaging (NASCI) on the coronary artery disease-reporting and data system (CAD-RADS) [25] was published. This way of reporting improved communication between the imager and the clinician and facilitated the collection of databases to further improve the classification of CAD. Key messages from this document are summarized in Table 1 and shown in Figure 3.

The guidelines also emphasize the importance of assessing and reporting plaque burden, high-risk features, and functional tests as additional “modifiers” to anatomic parameters in clinical decision-making [25].

### 3.9. Role of CCTA in Acute Coronary Syndromes

Acute chest pain is among the most common causes of admission to the ER worldwide. Prompt and standardized assessment and identification of acute coronary syndromes in this context is paramount.

The development of structured clinical decision pathways, supported by both 2021 AHA/ACC chest pain guidelines [36] and 2025 AHA/ACC acute coronary syndromes guidelines [37], have been shown to decrease unnecessary testing and admissions while maintaining high sensitivity for detection of acute myocardial injury and 30 day MACE [37]. Several large randomized trials have assessed the usefulness of CCTA in acute coronary syndromes (ACS), such as the ROMICAT II, ACRIN-PA, CT-COMPARE, and CT-STAT, leading to its inclusion in these decision pathways, particularly in the context of intermediate-risk patients with acute chest pain, although some low-risk patients with inconclusive ECG and/or troponin findings may also benefit from anatomic testing with CCTA [38]. Table 2 depicts current guideline recommendations of CCTA in acute and chronic coronary syndromes.

### 3.10. Evaluation of Cardiac Structure and Function

Besides the utility of Cardiac CT in assessing coronary artery disease, it is also a useful tool for evaluating cardiac structural disease, including pericardial, myocardial, and valvular heart disease.

#### 3.10.1. Pericardial Disease

Pericardial disease encompasses a diverse spectrum of congenital and acquired disorders, with highly variable clinical presentations that often necessitate targeted therapeutic approaches. In addition to primary pericardial pathology, the pericardium can be secondarily involved in a wide array of systemic and organ-specific diseases, including infectious, autoimmune, and neoplastic processes. Iatrogenic causes, such as those related to cardiac surgery or radiation therapy, also account for a substantial burden of pericardial-related complications [39].

Despite the well known role of echocardiography as first-line testing in the assessment of pericardial disease, a cardiac CT scan aids in the diagnosis in some pericardial abnormalities, particularly in patients with poor sonographic windows, and can even be superior in the assessment of certain characteristics (Table 3) [40].

**Figure 4:**
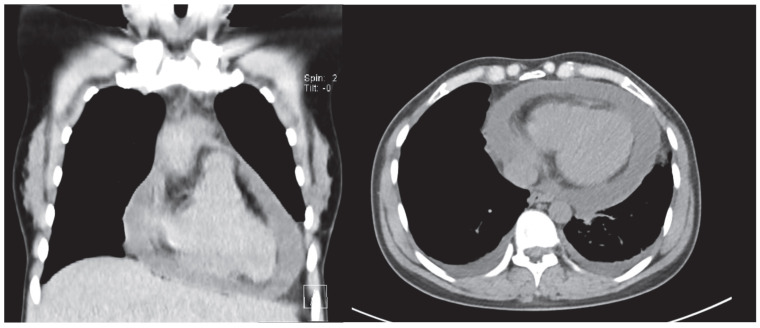
Non-contrast computer tomography showing severe pericardial effusion in a 36 year old female with unremarkable medical history. Pericardiocentesis was performed, diagnosing viral pericarditis.

Pericardial thickening and calcifications identified on cardiac CT are valuable in the assessment of patients with suspected constrictive pericarditis. Additionally, multiphasic imaging can aid in detecting pericardial adhesions in selected cases [39]. (Figure 4).

#### 3.10.2. Myocardial Characterization and Cardiac Function with CT

Cardiac CT is useful in assessing chamber morphology and size. End-diastolic images are used to measure the wall thickness of the left and right ventricles, as well as their overall size. The use of multidetector CT image acquisition (MDCT) enables the quantitative and qualitative assessment of left and right ventricular systolic function. Although there are variations in software between vendors, they typically employ retrospective ECG gating to measure left ventricular (LV) volume and left ventricular ejection fraction (LVEF) measurements. Evidence shows there is no significant difference between MDCT and cardiac MRI, the current gold standard for chamber morphology and ventricular ejection fraction [41,42].

The precision in assessing ventricular and valvular function strongly depends on the temporal resolution of the imaging modality used. Cardiac MRI, with its high temporal resolution (typically under 50 milliseconds), provides accurate quantification of left ventricular ejection fraction (LVEF), as well as valvular regurgitation. In contrast, conventional 64-slice single-source multidetector CT has a slower temporal resolution (around 150 milliseconds), which affects the ability to accurately image moving cardiac structures and often necessitates beta-blockade in patients with elevated heart rates, which may compromise functional accuracy [43]. The advent of dual-source CT has enhanced the temporal resolution to approximately 80 milliseconds, thereby improving the accuracy of LVEF evaluation and spatial resolution and often eliminating the need for beta blockers [42].

As temporal resolution continues to improve, CT is expected to allow an increasingly precise assessment of cardiac chamber function and valvular abnormalities. Despite MSCT’s ability to estimate LVEF reliably, it presents notable limitations: chief among them is notably the high radiation exposure associated with retrospective ECG-gated acquisitions that span both systole and diastole. In several studies included in a recent meta-analysis, the average effective radiation dose ranged from 8 to 14 mSv [42]; although prospective ECG-gated protocols are now available and significantly reduce radiation exposure, they typically capture only a single cardiac phase, limiting their utility for full-phase functional assessment [44].

#### 3.10.3. Valvular Heart Disease

In recent years, there have been significant developments in percutaneous techniques for replacement or repair in the field of valvular disease, which have changed current clinical practice worldwide. Along with major developments in these techniques, cardiac CT has emerged as one of the most important imaging techniques in preprocedural planning and postprocedural follow-up and assessment, and although echocardiography still remains the first-line imaging modality to assess valvular heart disease, transcatheter valve procedures depend significantly on CT imaging for planning the intervention, anticipating and identifying potential complications, and assessing prosthetic valve performance [45].

**Figure 5:**
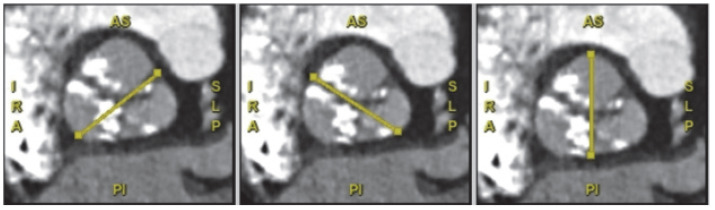
A 60 year old female with low flow low gradient severe aortic stenosis with severe valve calcification (2988 Agatston Units).

In the assessment of aortic stenosis, when there is uncertainty about the severity with echocardiography or when dobutamine stress echocardiography is inconclusive or contraindicated, non-contrast cardiac CT calcium scoring can be used to help confirm the severity of aortic stenosis (AS), as it offers a load-independent anatomical assessment. In men, calcium scores more than 2000 Agatston units, and more than 1200 in women, generally indicate severe aortic stenosis (AS) [46] (Figure 5).

**Figure 6:**
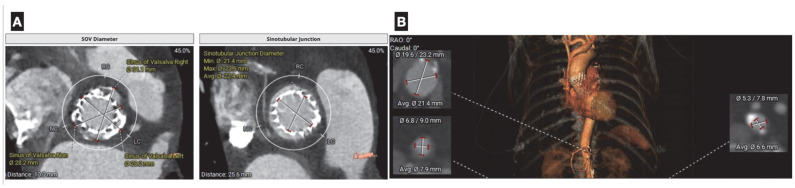
(**A**) Coronary computer tomography in planning of aortic valve-in-valve replacement. Measurement of aortic annulus size (diameter, area, and perimeter), is critical for selecting the appropriate prosthesis size to minimize the risks of paravalvular leak or annular rupture. Additional data, such as height of the coronary ostia, helps assess the risk of coronary obstruction during valve deployment. (**B**): Assessment of peripheral vasculature aids in the optimal selection of vascular accesses and minimizes the risk of vascular complications.

Furthermore, direct measurement of the aortic valve coaptation area defect can be achieved through planimetry at the leaflet tips. This technique also enables precise assessment of aortic regurgitation by examining valve closure during diastole. Prior to transcatheter valvular aortic replacement (TAVR), CCTA can offer precise measurements of not only the aortic annulus but of the entire aortic root and offers essential information in the assessment of vascular accesses [47] (Figure 6). The clinical impact of CT-based planning is supported by real-world evidence: Mylotte et al. reported that CT analysis frequently resulted in larger annular measurements than transesophageal echocardiography (TEE) alone, and that adherence to CT-based oversizing criteria was independently associated with a 21% lower incidence of paravalvular leak compared with TEE sizing (14% vs. 35%; *p* = 0.003) [48]. Similarly, in a prospective study of 266 patients, CT-based prosthesis sizing significantly reduced paravalvular leak rates (5.3% vs. 12.8%; *p* = 0.032) and in-hospital mortality (3.8% vs. 11.3%; *p* = 0.02) compared with sizing based on echocardiography and angiography [49]. Post-TAVR, cardiac CT is valuable in the evaluation of suspected prosthetic valve thrombosis, endocarditis, or structural deterioration, with characteristic findings such as hypoattenuated leaflet thickening (HALT) and reduced leaflet motion (HAM) providing important diagnostic information for targeted management. Likewise, Yin Ge et al. found that in a cohort of 80 candidates for mitral valve replacement who underwent pre-procedural cardiac CT, a substantial proportion were found to have unfavorable anatomy, resulting in the cancelation of the procedure [50].

**Figure 7:**
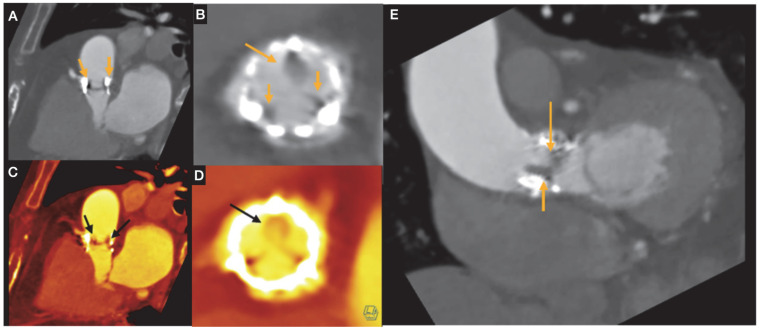
An 84 year old woman with a history of TAVI. She presents with dyspnea. Cardiac CT angiography revealed an aortic valve prosthesis with evidence of hypoattenuation and leaflet thickening (HALT), and absence of complete coaptation, with a calculated aortic valve orifice (AVo) area of 1.1 cm^2^ (prosthetic valve dysfunction). Top images (**A**,**B**) show the valve stent and thickened leaflet in an oblique view (**A**), and a short-axis view of the valvular structure during systole (**B**), with arrows indicating areas of hypoattenuation consistent with HALT. Bottom images (**C**,**D**) display color-coded reconstructions to enhance structural contrast. Arrows highlight the valvular stent. The bottom-right image (**D**) demonstrates lack of leaflet coaptation, contributing to valvular regurgitation (arrow). (**E**) depicts an oblique view of the valve during systole, showing absence of excursion of one of the leaflets as compared to the other (arrows).

Post-procedure cardiac CT information in assessing prosthetic valve function and structure. Following TAVR, cardiac CT imaging may be appropriate in cases where there is clinical concern for valve thrombosis, endocarditis, or structural deterioration of the prosthetic valve. Valve thrombosis is typically suspected when echocardiography shows increased transvalvular gradients, especially if accompanied by symptoms of aortic stenosis [47]. Signs of leaflet thrombosis include hypoattenuated thickening of the valve leaflets (HALT) and limited leaflet motion, referred to as hypoattenuation affecting motion (HAM). This thickening often has a crescent-like shape, and is more pronounced at the base of the leaflet (Figure 7). These findings should be documented in terms of location, extent, and thickness, and whether leaflet motion is restricted should also be noted. Differentiation between true HALT and beam hardening is important; true hypoattenuation is characterized by consistent localization within the leaflet tissue and correlates with restricted leaflet motion, whereas beam hardening artifacts typically appear as irregular streaks adjacent to the metallic stent and do not correspond to leaflet dysfunction.

Valve thrombosis classification is based on the percentage of sinus of Valsalva thrombosis occupation seen in the MSCT as HALT, using grades 1 to 4, defined as >25%, 26–50%, 51–75% and >75%, respectively.

Most HALT cases with restricted motion are asymptomatic. Oral anticoagulation has been linked to a lower risk of developing HALT or HAM and can help reverse thickening when it occurs. However, the benefit of treating subclinical leaflet thrombosis remains uncertain, and it is unclear whether this approach reduces the risk of future valve degeneration. In a large real-world population undergoing TAVR and routine CTA screening 30 days afterward, HALT was detected in 12% of cases and was independently linked to increased long-term mortality [51].

In mitral valve replacement, left ventricular outflow tract (LVOT) obstruction is a potential complication of transcatheter mitral valve replacement (TMVR). During TMVR, a patient’s native outflow tract can be altered, with the resulting LVOT sometimes referred to as the “neo-LVOT”. Preprocedural CT imaging, which can simulate the neo-LVOT, is useful for predicting the risk of obstruction. Recently, a study of patients undergoing TMVR found that a neo-LVOT area smaller than 1.7 cm^2^ at the end-systole posed a high risk of obstruction [52]. Other anatomical characteristics analyzed with MSCT are the presence and conformation of calcium at the level of the annulus (MAC), mitral anterior leaflet length to recognize high-risk of LVOT obstruction, and annulus diameter to choose prosthesis size.

#### 3.10.4. Aortic Diseases

**Figure 8:**
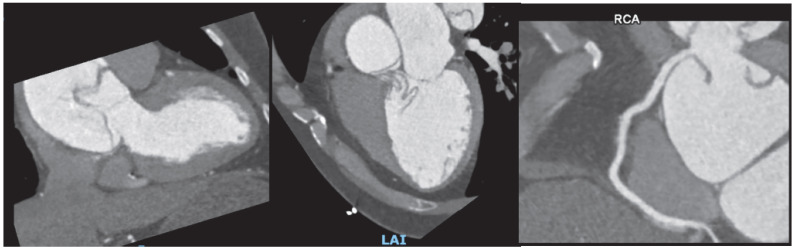
A 56 year old man presented to the emergency department with chest pain and ST elevation in right precordial leads. POCUS showed an aortic dissection flap with severe aortic regurgitation. This was confirmed on CCTA establishing right coronary involvement.

Cardiac CT offers important information in the evaluation of aortic syndromes, including aortic dissection, intramural hematoma, and penetrating aortic ulcer. Its high spatial and temporal resolution, combined with rapid acquisition time, makes CT angiography (CTA) the first-line imaging modality in emergency settings. It enables precise visualization of the intimal flap, false lumen, branch vessel involvement, and extension of the dissection, which are critical for surgical or interventional planning. (Figure 8) [53].

#### 3.10.5. Congenital Heart Disease

**Figure 9:**
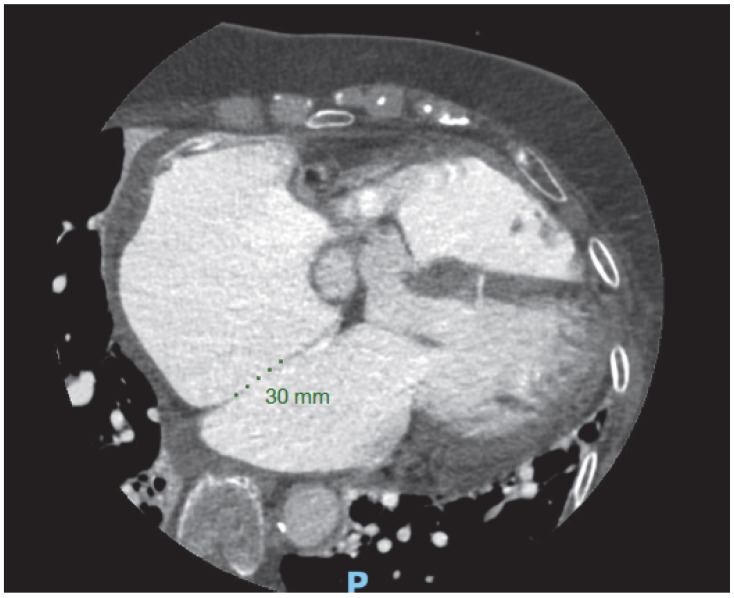
Coronary computer tomography showing a 30 mm atrial septal defect and a non-significant ventricular septal defect in an adult patient with non-relevant medical history who presented to the ER with dyspnea and atrial fibrillation.

In the assessment of congenital heart disease, cardiac computed tomography (CT) has a major role in anatomical assessment and surgical planning. Its ability to provide high-resolution, three-dimensional visualization of cardiovascular structures is especially useful in cases involving anomalous pulmonary venous return, complex aortic arch anomalies, coronary artery anomalies, and post-surgical repairs [54]. Furthermore, CT is valuable for long-term surveillance in postoperative CHD patients, including assessment of surgical grafts and conduits as well as in the longitudinal evaluation of the structural repercussions of hemodynamic alterations in this patient population [55] (Figure 9).

## 4. Other Applications

In atrial fibrillation, pulmonary vein anatomy assessment is important in pre-procedural planning of ablation techniques, and while not first-line testing, it can aid in the identification of left appendage thrombus. A filling defect observed in the left atrial appendage could simply indicate sluggish blood flow; therefore, delayed contrast-enhanced imaging may be required to distinguish it from an actual thrombus.

In acute pulmonary embolism, CCTA is an essential tool, as it not only establishes the diagnosis with great precision but also provides important prognostic information by assessing signs of right ventricular (RV) failure, a key marker of severity in this context. Two validated imaging markers include an increased RV to left ventricular (LV) diameter ratio (RV/LV > 1), which suggests RV dysfunction, and a pulmonary artery to ascending aorta diameter ratio (PA/Ao > 1), which is associated with elevated pulmonary pressures. These findings are strongly linked to adverse outcomes and can guide clinical decision-making in patients with acute PE [56].

### Cardiac Allograft Vasculopathy

Cardiac allograft vasculopathy (CAV) is a very well acknowledged complication presenting after five years in up to 30–50% of patients undergoing heart transplantation, and it is a leading cause of allograft failure and death in heart transplant receivers [57]. Although both can coexist in a single patient, several key differences in pathophysiology and clinical presentations between CAV and atherosclerotic CAD should be noted; CAV can develop within one to two years after heart transplantation, progresses variably, and unlike focal, calcified native atherosclerosis has a more extensive distribution with CAV-related wall thickening typically affecting the whole length of the coronaries in a concentric fashion (concentric intimal hyperplasia). Another key difference between CAV and atherosclerotic CAD is that CAV lesions are typically non-calcified, whereas atherosclerotic CAD commonly exhibits substantial calcium deposition within plaques. In 2010, a consensus paper commissioned by the International Society of Heart and Lung Transplantation (ISHLT) board established a standardized CAV definition and classification system, and designated invasive coronary angiography (ICA) (with or without the use of intravascular ultrasound) as the accessible gold standard for surveillance with a recommendation of serial assessment every one to two years [58]. The rise in new CT technologies (such as photon counting CT) and protocols (with reduced radiation), along with evidence of excellent diagnostic performance, has spurred calls for updated recommendations. CCTA offers high sensitivity (up to 97%), specificity (up to 97%), and excellent negative predictive value (NPV up to 99%) for detecting significant stenosis in heart transplant recipients [59]. Beyond anatomical assessment, CCTA allows functional evaluation through CT-derived fractional flow reserve (FFRct) and facilitates quantitative plaque analysis, enabling earlier detection of diffuse and concentric intimal thickening that is typical of CAV. While considerations remain regarding radiation exposure, contrast use, and limited microvascular assessment, CCTA’s comprehensive, multiparametric capabilities position it as a powerful tool for routine CAV monitoring and risk stratification in heart transplant follow-up, particularly compared to ICA, which is not devoid of complications, typically conveying greater radiation and contrast exposure [59].

## 5. Future Directions

Future directions in CCTA are rapidly advancing, particularly with the integration of artificial intelligence (AI), which enhances image acquisition, reconstruction, and diagnostic interpretation by reducing noise and improving efficiency [60]. AI algorithms are also being developed to detect coronary stenoses, assess plaque characteristics, and even predict major adverse cardiovascular events based on CCTA data [61]. Myocardial viability assessment is another growing application, with studies showing that dynamic CT perfusion imaging and delayed iodine enhancement can identify viable versus non-viable myocardium, offering an alternative to nuclear or MRI-based techniques.

As discussed in previous section of this article, one of the major limitations of CCTA is the overestimation of stenosis severity in comparison to coronary angiography, particularly in the assessment of highly calcific plaques [3].

This is due to several limitations inherent to conventional energy-integrated detector CT imaging (EID-CT), a technology that uses layered scintillators to transform X-ray beams to visible light, and then, a photodiode array converts this light into electrical signals [62]. This layered and dual phase design leads to key shortcomings, chief among them being limited spatial resolution, which causes a voxel in the CT image containing both high-density calcium and lower-density surrounding tissues, resulting in an average high Hounsfield Unit (HU) value to the entire voxel, leading to size overestimation of calcium deposition [63]. Secondly, conventional energy-integrating detectors measure the total energy deposited by all incoming photons, not their individual energies. This lack of energy discrimination leads to the loss of spectral information, precluding fine discernment between calcium and soft plaque/normal tissue, and resulting in varying degrees of “blooming” artifacts that overshadow the coronary lumen [63].

New CT technologies have been developed to overcome these limitations; among these, ultra high-resolution photon-counting detector CT (UHR PCD-CT) represents a major advancement. Unlike EID-CT, photon-counting detectors register and count individual X-ray photons and measure their energy, enabling true spectral imaging at the detector level without the need for dual-source or dual-layer hardware [64]. This technology offers substantially improved spatial resolution, reduced electronic noise, and intrinsic spectral separation, allowing for more accurate differentiation between calcified plaque, non-calcified plaque, and lumen; these features translate into less blooming artifacts from calcifications, sharper vessel edge definition, and improved quantification of stenosis severity [62]. Additionally, the higher contrast-to-noise ratio of PCD-CT enables lower radiation dose protocols and better visualization of small cardiac structures, making it a promising tool for more precise, noninvasive coronary artery assessment in both heavily calcified and complex atherosclerotic diseases [65]. A prospective study compared coronary stenosis degrees and corresponding CAD-RADS [66] categories between UHR PCD-CT and EID-CT scans and assessed accuracy against an invasive coronary angiogram, CT angiography using both ultrahigh-spatial-resolution photon-counting detector CT (PCD-CT), and conventional energy-integrating detector CT (EID-CT) within 30 days, then had invasive quantitative coronary angiography (QCA) for validation. Among 278 plaques across 49 patients (202 calcified, 51 partially calcified, 25 non-calcified), the study found that PCD-CT measured a significantly lower percentage of diameter stenosis (PDS) compared to EID-CT for both calcified (45.1 ± 20.7% vs. 54.6 ± 19.2%; *p* < 0.001) and partially calcified lesions (44.3 ± 19.6% vs. 54.9 ± 20.0%; *p* < 0.001), while non-calcified plaque assessments were essentially identical (≈39%). This reduction in overestimation led to a remarkable 49% of patients being reclassified to a lower CAD-RADS category when using PCD-CT. Importantly, in the subset of patients who underwent invasive angiography, PCD-CT stenosis measurements more closely agreed with QCA (mean difference ≈ 7.3%, limits of agreement −10.7% to 25.2%) versus the wider bias observed with EID-CT (mean difference ≈ 17.4%, limits −6.9% to 41.7%). These findings underline that UHR-mode PCD-CT not only reduces blooming artifacts but also enhances quantitative accuracy in calcified coronary lesions and meaningfully alters clinical stenosis grading [66].

Percutaneous coronary intervention planning is a novel topic, where specific characteristics assessed by CCTA, such as plaque calcium burden, plaque length and diameter, angiographic work projections; vessel ostium localization in post-coronary artery bypass grafting patients can reduce time, contrast and radiation in these interventional procedures [67].

These advances situate the CCTA not just as an anatomical tool but also as a comprehensive modality capable of functional and tissue-level cardiac assessment, showing that this tool has not reached its maximum potential [67]. The future of cardiac CT is currently focused on delivering significantly lower radiation doses through advanced acquisition techniques and detector technologies, while improving cost-effectiveness by incorporating machine learning and AI protocols, enabling faster, more accurate, and widely accessible cardiovascular assessment.

## 6. Conclusions

Cardiac computed tomography has evolved from a niche tool for coronary calcium scoring into a comprehensive, multiparametric imaging modality that integrates anatomical, functional, and prognostic information in a single examination. Coronary artery calcium quantification remains a robust, widely available method for risk stratification, guiding preventive strategies, and refining cardiovascular risk assessment across diverse patient populations. Coronary CT angiography provides high-resolution characterization of coronary anatomy and plaque morphology, identifies high-risk features predictive of acute coronary syndromes, and, when complemented by CT-derived fractional flow reserve or perfusion imaging, enables precise assessment of lesion-specific ischemia to inform revascularization decisions.

Beyond coronary disease, cardiac CT is indispensable for structural heart disease evaluation; preprocedural planning of transcatheter interventions; assessment of pericardial, myocardial, valvular, aortic, and congenital abnormalities; and risk stratification in acute coronary syndromes and pulmonary embolism. Continuous technological innovations—including dual-energy imaging, photon-counting detectors, and artificial intelligence—are enhancing spatial resolution, reducing radiation exposure, minimizing blooming artifacts, and expanding diagnostic capabilities toward tissue characterization and physiologic assessment.

Clinically, cardiac CT’s expanding role supports earlier diagnosis, individualized treatment planning, and improved outcomes through better risk prediction and therapeutic targeting. From a research perspective, it provides a versatile platform for longitudinal studies on disease progression, therapy response, and prognostic modeling.

Despite major advances in scanner technology, image reconstruction, and dose-reduction algorithms, cardiac computed tomography (CT) continues to face important challenges. Radiation exposure, although significantly reduced with prospective gating, iterative reconstruction, and novel photon-counting techniques, remains a consideration in some scenarios, chief among them being younger patients or those requiring repeated imaging. Additionally, the high cost of advanced CT systems, specialized software, and expert interpretation can limit accessibility, especially in resource-constrained settings. These factors underscore the need for judicious patient selection and ongoing innovation to balance diagnostic benefit with safety and cost-effectiveness.

## Figures and Tables

**Table 1:** CAD-RADS Categories (Adapted from Cury et al., 2022) [25].

Category	Degree of Coronary Stenosis	Further Investigations	Management Considerations
0	0%—No CAD	None needed	Assess non-atheroesclerotic causes of symptoms. General preventive measures
1	1–24%—Minimal non-obstructive CAD	None needed	Assess non-atheroesclerotic causes of symptoms. Risk factor modification, consider preventive pharmacotherapy
2	25–49%—Mild non-obstructive CAD	None needed	Assess non-atheroesclerotic causes of symptoms. Risk factor modification, consider preventive pharmacotherapy
3	50–69%—Moderate stenosis	Functional assessment is recommended	Prior recommendations + other treatments, including antianginals per guideline-directed care
4A	70–99% single vessel stenosis	Invasive coronary angiography or functional assessment are recommended	Prior recommendations + other treatments, including antianginals per guideline-directed care
4B	Left main 50% or 3-vessel obstructive (70%) disease	Invasive coronary angiography is recommended	Revascularization should be considered + other treatments, including antianginals per guideline-directed care
5	100%—Total occlusion	Consider ICA, functional and/ or viability assessment	Revascularization should be considered after viability/individual assessment + other treatments, including antianginals per guideline-directed care
N	Non-diagnostic study	Additional/alternative evaluation may be needed	Additional/alternative evaluation may be needed

**Table 2:** Guideline recommendations of CCTA in acute and chronic coronary syndromes.

Clinical Scenario	American Heart Association/American College of Cardiology	European Society of Cardiology
Acute Coronary Syndromes	**Class I**: CCTA may be considered for patients with lower risk NSTE-ACS as a non-invasive risk stratification, as part of a selective invasive strategy (1).	**Class IIA**: Incorporating CCTA or a non-invasive stress imaging test as part of the initial workup in patients with suspected ACS, non-elevated (or uncertain) hs-cTn, no ECG changes, and no recurrence of pain should be considered. **Class IIIB**: Routine, early CCTA in patients with suspected ACS is not recommended.
Chronic Coronary Syndromes	**Class IIA:** For patients with CCD and a change in symptoms or functional capacity that persists despite GDMT, and who have had previous coronary revascularization, CCTA is reasonable to evaluate bypass graft or stent patency (for stents ≥ 3 mm). **Class IIA**: For selected patients with suspected non-ischemic cardiomyopathy, CCTA may be considered as an initial diagnostic strategy.	**Class I, Level B**: CCTA is recommended as a first-line test for patients with low to intermediate pre-test probability of CAD.

**Table 3:** Comparison between echocardiography and cardiac CT in the assessment of pericardial pathology.

Pericardial Feature/Condition	Echocardiogram	Cardiac CT
**Pericardial Thickness**	Limited for precise measurement; may suggest thickening indirectly	High spatial resolution; precise measurement of pericardial thickness
**Pericardial Calcifications**	Poor sensitivity; typically, not visualized	Excellent for detecting and quantifying calcifications
**Pericardial Inflammation**	Can suggest inflammation (e.g., fibrin strands, increased echogenicity)	Limited unless contrast enhancement shows indirect signs
**Pericardial Adhesions**	Indirect signs (e.g., septal bounce), but not definitive	Can identify adhesions through restricted motion in multiphasic imaging
**Pericardial Effusion Detection**	High sensitivity; excellent for real-time detection	Good sensitivity, especially for loculated effusions
**Pericardial Masses**	Can detect large masses, but limited soft-tissue characterization	Superior soft-tissue contrast; good for mass localization and extent

## Data Availability

No new data were created or analyzed in this study. Data sharing is not applicable to this article.
